# Study on the Identification and Detection of Walnut Quality Based on Terahertz Imaging

**DOI:** 10.3390/foods11213498

**Published:** 2022-11-03

**Authors:** Jun Hu, Hongyang Shi, Chaohui Zhan, Peng Qiao, Yong He, Yande Liu

**Affiliations:** 1 School of Mechanical and Electrical Engineering, East China Jiaotong University, Nanchang 330013, China; 2 School of Mechanical Engineering, Zhejiang University, Hangzhou 310027, China

**Keywords:** walnut, quality inspection, qualitative discriminant analysis, fullness, mildew

## Abstract

Objective: Walnuts have rich nutritional value and are favored by the majority of consumers. As walnuts are shelled nuts, they are prone to suffer from defects such as mildew during storage. The fullness and mildew of the fruit impose effects on the quality of the walnuts. Therefore, it is of great economic significance to carry out a study on the rapid, non-destructive detection of walnut quality. Methods: Terahertz spectroscopy, with wavelengths between infrared and electromagnetic waves, has unique detection advantages. In this paper, the rapid and nondestructive detection of walnut mildew and fullness based on terahertz spectroscopy is carried out using the emerging terahertz transmission spectroscopy imaging technology. First, the normal walnuts and mildewed walnuts are identified and analyzed. At the same time, the image processing is carried out on the physical samples with different kernel sizes to calculate the fullness of the walnut kernels. The THz image of the walnuts is collected to extract the spectral information in different regions of interest. Four kinds of time domain signals in different regions of interest are extracted, and three qualitative discrimination models are established, including the support vector machine (SVM), random forest (RF), and k-nearest neighbor (KNN) algorithms. In addition, in order to realize the visual expression of walnut fullness, the terahertz images of the walnut are segmented with a binarization threshold, and the walnut fullness is calculated by the proportion of the shell and kernel pixels. Results: In the frequency domain signal, the amplitude intensity from high to low is the mildew sample, walnut kernel, and walnut shell, and the distinction between walnut kernel, shell samples, and mildew samples is high. The overall identification accuracy of the aforementioned three models is 90.83%, 97.38%, and 97.87%, respectively. Among them, KNN has the best qualitative discrimination effect. In a single category, the recognition accuracy of the model for the walnut kernel, walnut shell, mildew sample, and reference group (background) reaches 94%, 100%, 97.43%, and 100%, respectively. The terahertz transmission images of the five categories of walnut samples with different kernel sizes are processed to visualize the detection of kernel fullness inside walnuts, and the errors are less than 5% compared to the actual fullness of walnuts. Conclusion: This study illustrates that terahertz spectroscopy detection can achieve the detection of walnut mildew, and terahertz imaging technology can realize the visual expression and fullness calculation of walnut kernels. Terahertz spectroscopy and imaging provides a non-destructive detection method for walnut quality, which can provide a reference for the quality detection of other dried nuts with shells, thus having significant practical value.

## 1. Introduction

Walnuts, along with almonds, cashews, and hazelnuts, are the world-renowned “four dried nuts” with rich nutritional value. The plumpness of the walnut is a key factor; if the walnut kernel is not full, it is possible it lacked nutrition during the growth process, which seriously affects the quality of the walnut. Walnut kernels are mainly processed in China, and the main products are walnut food, walnut oil, and walnut health products, which are favored by more and more consumers and have great edible and commercial value [1]. During storage, walnuts are prone to oil oxidation, protein denaturation, texture drying, color browning, flavor loss, internal insect damage, and kernel mildew [2]. Inadvertent consumption of deteriorated walnuts can cause diarrhea, vomiting, and even cancer; thus, there is an urgent need for effective, non-destructive detection technology to monitor their quality in real time. Fruit fullness and mildew will affect the quality of walnuts, but walnuts have hard shells, which greatly hinders the effective extraction of its internal information, which discourages previous non-destructive quality detection methods. Therefore, it is of great significance to carry out a study on rapid, non-destructive detection of walnut internal quality for the realization of the walnut quality, safety monitoring, and the healthy development of the walnut industry.

In recent years, various imaging technologies such as X-ray imaging, infrared imaging, and hyper-spectral imaging have been used for the detection of the internal quality of food. Chuang C L et al. [3] developed an automatic detection device for internal pests of agricultural products based on an x-ray with the main detection targets being fruits, such as peaches and guavas. The results showed that the device can accurately locate internal pests in the fruits with a localization accuracy of 94%. Long Y et al. [4] applied a line scanning Raman hyper-spectral imaging system to extract corn mildew texture features and established an optimal detection model. The results showed that the mildew of maize kernels can be effectively detected with Raman hyper-spectral technology. Jiang H Z et al. [5] applied the hyper-spectral imaging system to identify Camellia oil tea fruits with different degrees of mildew, and the CARS-PLS-DA model showed the best performance. The overall results showed that the hyper-spectral imaging technology provides a solution for detecting the degree of natural mildew of Camellia oil tea fruits. Each detection technology has its own advantages and disadvantages, and a single technology cannot meet all the requirements of the food industry for the analysis of complex foreign bodies and food substances. For instance, X-ray imaging is difficult to detect foreign bodies with a density lower than that of the food matrix, and its ionizing radiation is harmful to both the experimenter and the sample [6]. Although the information obtained with hyper-spectral imaging is relatively more comprehensive, it is difficult to penetrate the samples with hard shells. Due to the thick shell of walnuts, it is difficult to carry out the detection of the actual walnut. In order to meet the urgent demand for safe and high-quality food, more effective technologies are needed to achieve the internal quality detection of food.

As an emerging non-destructive detection method, terahertz spectroscopy imaging technology has dual characteristics of microwave and infrared technologies, which can penetrate and interact with many commonly used materials. Terahertz spectroscopy and imaging technology has great application potential in the field of food quality detection due to its penetrability, fingerprint identification, and safety. Jiang Y et al. [7] used terahertz time domain spectral reflectance imaging technology to detect foreign bodies in wheat grains at different depths, and terahertz images were processed using image preprocessing and threshold segmentation algorithms to achieve the detection of foreign bodies in flour. Wang C et al. [8] realized the detection of metal foreign bodies in sausages by terahertz imaging technology. Jun Hu et al. [9] used terahertz imaging technology to detect common foreign bodies in milk powder, such as polymer materials (PP, PVC, PE) and metal gaskets. Sun X D et al. [10] used terahertz time-domain transmission imaging system to scan the terahertz data of sunflower seeds and segmented the image with binary value. The results showed that terahertz technology is able to detect the fullness of sunflower seeds. Qi S Y et al. [11] realized the identification of normal walnuts, insect-infested walnuts, and mildew walnuts using a powder compression method. Xie L et al. [12] detected endogenous foreign bodies in walnut kernels by comparing the typical absorption spectra of walnut kernels and walnut shells at different concentrations, and they identified shell contamination in walnut kernels effectively using terahertz imaging technology.

The detection of actual walnuts is difficult due to their thick shells. Therefore, there are few studies that have been carried out on the detection of the fullness of the walnuts. In this paper, terahertz time-domain spectroscopy transmission imaging technology is used to study the mildew and fullness of walnuts. First, the image information of different types of walnuts is collected, and the spectral data of the walnut shell, walnut kernel, mildew sample, and reference group in four different regions of interest are extracted. Three qualitative detection models of SVM, RF, and KNN are established to explore the optimal model through the accuracy rate and identify the normal walnuts and mildew walnut. Second, the visualization of walnut fullness is studied. The transmission imaging of walnut samples with different fullness is carried out, and the image is segmented using a binarization threshold. The proportion of the walnut kernel and walnut shell is calculated with pixels to realize the detection and calculation of walnut fullness.

## 2. Materials and Methods

### 2.1. Sample Preparation

**Figure 1:**
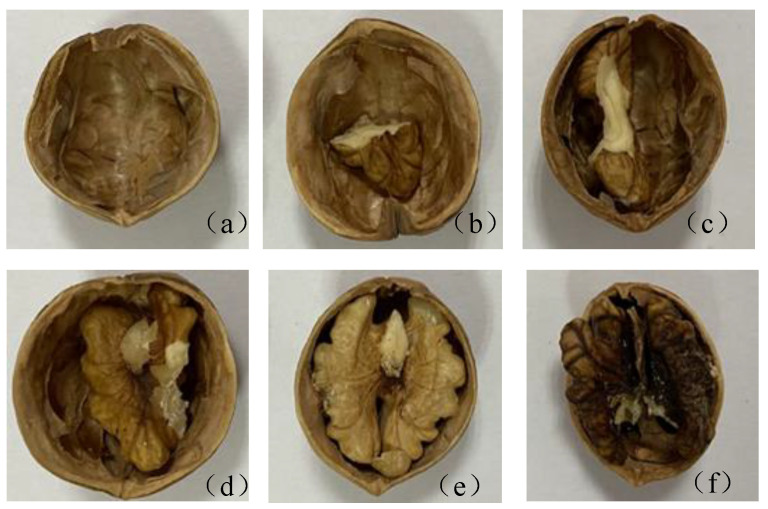
Internal images of walnut samples. (**a**) Empty shell; (**b**) 1/4 Walnut kernel; (**c**) 1/2 Walnut kernel; (**d**) 3/4 Walnut kernel; (**e**) Plump walnuts; (**f**) Mildewed kernel.

The walnuts used in this experiment were purchased from a large supermarket and originated from Aksu, China. Half of them were kept in a dry place for normal storage, and the other half were placed in a humid environment to make them naturally mildewed. After a period of time, all the walnuts were peeled off to observe whether the kernels were normal. Five normal walnuts were randomly selected and the kernels were processed into different sizes, and one kernel with a large mildew area was selected as the mildew sample. Figure 1 shows the images of peeled walnuts. The images are (a), (b), (c), (d), and (e) according to the kernels from small to large, while (f) is the mildewed kernel. Terahertz imaging was performed on these six types of samples, and due to the limitation of the detection platform, terahertz image information of each walnut was collected separately. Because of the spherical shape of walnuts, the half-nuts were used for the experiment to facilitate sample fixation.

### 2.2. THz-TDS Spectroscopy System

**Figure 2:**
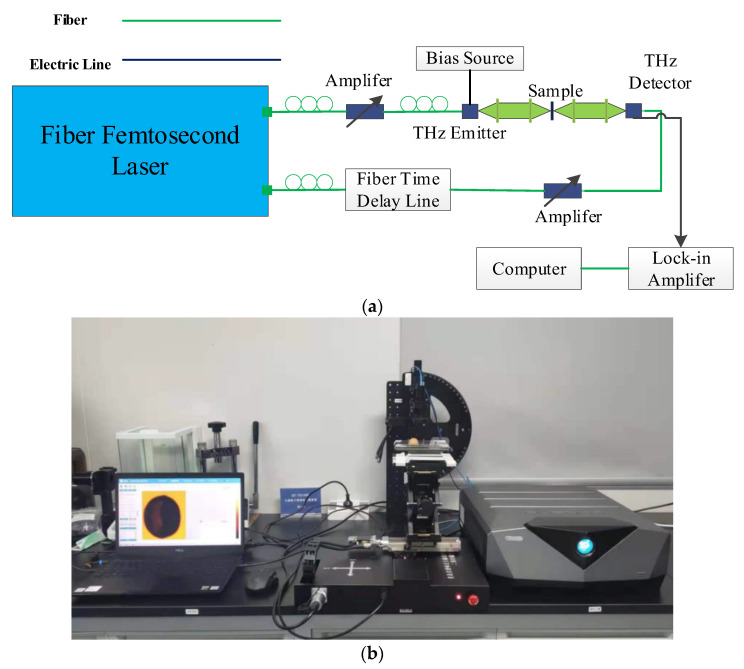
(**a**) Schematic diagram of QT-TS1000 terahertz spectral imaging system. (**b**) Physical picture of QT-TS1000 terahertz device.

The terahertz image scanning system used in this experiment is a three-dimensional imaging system (Model: QT-TO1000, Qingdao Qingyuan Fengda Terahertz Technology Co., Ltd., Qingdao, China). Schematic diagram is shown in Figure 2a.The femtosecond laser generates femtosecond pulses, which are divided into pump light and probe light. The pump light enters the terahertz emitter and produces terahertz radiation. The terahertz pulse emitted by the transmitter reaches the sample and is reflected through the sample surface. The terahertz spectrum carrying sample information is focused and received by the detector, Physical picture of terahertz device is shown in Figure 2b. In this experiment, the terahertz frequency is 0.1–4.0 THz, the maximum scanning area is 100 × 100 mm^2^, the maximum detection thickness is 9 mm, and the maximum imaging speed is 60 pixel/s.

During the experiment, the ambient temperature of the laboratory was 24 ± 0.5 °C, and the humidity was lower than 10%. After preheating for half an hour for the equipment to stabilize, the terahertz transmission images of the walnut samples were acquired using the above equipment. The walnuts were placed on a mobile platform with the shelled side facing upward and moved through the X-Y platform, and the terahertz imaging camera was controlled using software settings to obtain the transmission images of the walnuts using point scanning. Considering that the polyethylene plate (PE plate) has basically no absorption in the terahertz band, a PE plate with a thickness of 1 mm was used as the supporting platform for the samples in the experiment.

### 2.3. THz Spectroscopy Detection System for Walnut Samples

The original data of the sample were obtained based on the time domain signal. In order to ensure the stability of the system and the robustness of the model, the frequency domain information of the sample could be extracted using the traditional fast Fourier transform. Based on the optical parameter extraction model proposed by Timothy [13] and Duvillaret et al. [14], the frequency spectrum distribution of the terahertz pulse was obtained with a fast Fourier transform (FFT). It can be expressed as Formula (1), where A(ω) represents the amplitude of the electric field, φ(ω) is the phase of the electric field, and E(t) is the terahertz time domain waveform.

(1) $$E\left(\omega \right)=A\left(\omega \right)e^{-i\varphi \left(t\right)}=\int dtE\left(t\right)e^{-i\omega t}$$

wherein, A(ω) is amplitude of the pulse signal, φ(ω) is the phase of the signal, and E(t) is the time domain light wave signal. The absorption coefficient α(ω) and refractive index n(ω) of each sample and reference sample are expressed as the following:

(2) $$\alpha \left(\omega \right)=\frac{2}{d}\ln\left\{\frac{4n\left(\omega \right)}{A\left(\omega \right){\left[1+n\left(\omega \right)\right]}^{2}}\right\}$$

(3) $$n\left(\omega \right)=1+\Delta \phi \left(\omega \right)\cdot \frac{c}{\omega d}$$

wherein, d is the sample thickness and c is light speed in a vacuum.

### 2.4. Terahertz Spectrum and Image Processing

**Figure 3:**
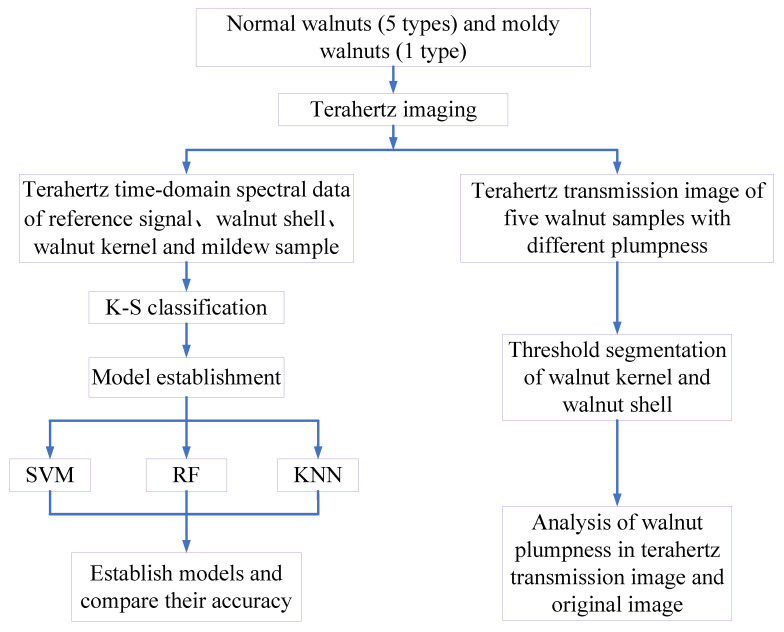
Processing flow for visual analysis of moldy walnuts and fullness.

In order to facilitate the analysis of terahertz spectral data and images, a chemometrics algorithm and binarization were used for processing. Figure 3 shows the flow chart of data and image processing. The QT-TO1000 terahertz system was used to acquire the terahertz spectral images of the reference signal, walnut shell, walnut kernel, and mildew sample, and the spectral information for the region of interest was extracted. The terahertz time-domain spectral data was divided into a calibration set and prediction set according to the ratio of 3:1 using the K-S algorithm. The calibration set was used to establish the model with cross validation, and the prediction set was used to verify the established model. Finally, the established model was evaluated using the model evaluation index. The data acquired was processed and analyzed using matlab2018a and Unscrambler10.4. The terahertz transmission image and the original image were binarized, and the plumpness of the walnut was characterized by calculating the proportion of walnut kernel pixels in the walnut shell.

### 2.5. Algorithm Principle of Qualitative Discrimination Model

(1) The support vector machine (SVM) algorithm is a classical model that maps the input vector from a low-dimensional sample space to a high-dimensional or infinite-dimensional one, thus converting nonlinear classification into a linear one [15]. When applied to spectral data processing, a qualitative discriminant model between spectral data and classification variables is established. SVM is defined as the following:

(4) $$\left\{ \begin{array}{l} \underset{\omega ,b,\xi }{min}J_{P}\left(\omega ,\xi \right)=\frac{1}{2}\omega^{T}\omega +\gamma \sum_{K=1}^{N}\xi_{k}\\ s.ty_{k}\left[\omega^{T}\varphi \left(x_{k}\right)+b\right]\geq 1-\xi_{k},k=1,\ldots ,N\\ \xi_{k}\geq 0,k=1,\ldots ,N \end{array}\right.$$

(2) The random forest (RF) algorithm is a machine learning method based on a decision tree. Similar to the forest in nature, the RF algorithm is composed of trees whose basic constituent units are decision trees, in which each decision tree is independent from each other [16]. The number of decision trees is established based on the number of variability between sample subsets, and the final judgment is obtained with a voting mechanism.

(3) The k-nearest neighbor (KNN) classification is used for modeling and model prediction. The core idea is that if most of the k-nearest samples in the feature space of a sample belong to a certain category, then this sample also belongs to this category [17]. When the two selected samples are similar to each other, the Euclidean distance is used as the distance metric, which is defined using Formula (5).

(5) $$L_{p}=\left(x_{i},x_{j}\right)={\left({\sum_{i=1}^{n}\left|x_{i}{}^{\left(l\right)}-x_{j}{}^{\left(l\right)}\right|}^{p}\right)}^{\frac{1}{p}}$$

### 2.6. Image Binarization Processing

Image binarization is the process of obtaining a binarized image with 256 brightness levels of grayscale images that can still reflect the overall and local characteristics of the image with appropriate threshold selection [18]. In this study, Matlab2018b (The MathWorks Inc., Natick, MA, USA) software was used to binarize the image, and the threshold value was adjusted so that the image with the threshold value as a demarcation can separate the full and empty areas of the walnuts with maximum difference to form a black and white image. The binarization of images is conducive to the subsequent identification and acquisition of the ratio of pixels in the shell and kernel regions, thus enabling the calculation of the walnut fullness.

### 2.7. Model Evaluation Method

In the three qualitative discriminant models established in this paper, the accuracy of the prediction model was used to evaluate the quality of the model. The calculation formula for the accuracy is shown as Formula (6).

(6) $$p=\frac{y_{i}}{y}\times 100\%$$

wherein $y_{i}$ is the correct number of model prediction classifications, and y is the total number of prediction set samples.

In this paper, the fullness of the walnut was calculated using the ratio of the number of walnut kernel pixels to the number of walnut shell pixels, and the fullness was calculated with Formula (7).

(7) $$p=\frac{x_{i}}{x}\times 100\%$$

wherein $x_{i}$ is the number of walnut kernel pixels, and x is the number of walnut shell pixels.

## 3. Results and Discussion

### 3.1. THz Spectral Feature Analysis

**Figure 4:**
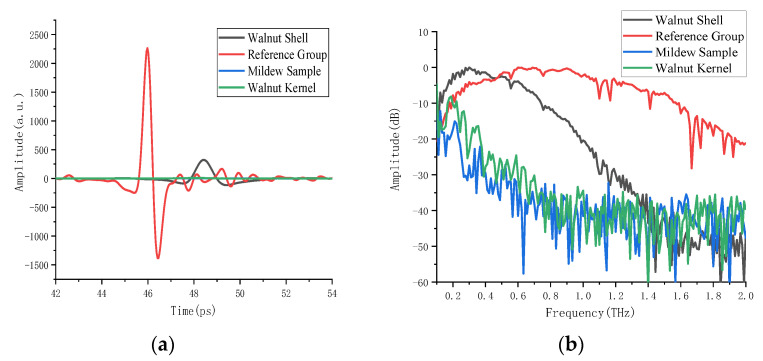
Terahertz time-domain and frequency-domain spectrum of walnut shell, walnut kernel, mildew samples and reference signal. (**a**) Time-domain signal (**b**) Absorption coefficient.

The THz images of walnut samples are acquired using a terahertz imaging system, and the time domain spectra of regions of interest in the background, walnut kernel, walnut shell, and mildew samples are extracted. In order to improve the robustness of the model, about 600 points of interest are extracted in each region. Figure 4 shows the THz spectrum of the walnut samples, in which Figure 4a is the time domain signal. The time domain spectrum in the range of 42–54 ps is selected for analysis to reduce the noise interference. It can be seen from Figure 4 that, compared with the reference signal, the time-domain signals of the walnut kernel, walnut shell, and mildew samples have a certain time delay, and the amplitude has a significant attenuation. Among them, the absorption intensity of the walnut kernel is similar to that of the mildewed sample. The frequency domain signal is obtained with a Fourier transform of the terahertz time domain signal. Figure 4b is the frequency domain signal. It can be seen that between 0.5–2.0 THz, the amplitude intensity of the signal from high to low is as follows: reference signal, walnut shell, walnut kernel, and mildew sample. The reference region does not absorb terahertz, so the amplitude of the reference signal is the strongest. Because the main component of a walnut shell is wood fiber, the absorption of the terahertz wave is weak, so the amplitude of this walnut shell sample is higher. The walnut kernel and mildew samples contain a lot of oil and water, and they have a strong absorption of terahertz waves; therefore, their amplitude intensity is the lowest. Due to the different time-domain and frequency-domain signals of the walnut shell, walnut kernel, and mildew samples, this provides a theoretical basis for the quality identification of walnuts using terahertz spectroscopy combined with a pattern recognition method.

### 3.2. Establishment of SVM Qualitative Determination Model for Terahertz Spectrum in Different Regions of Interest of Walnuts

In the four types of regions, 2445 points of interest are randomly selected, and the terahertz time-domain spectra of each pixel are extracted. The time-domain signals of 2250 terahertz bands in the full spectrum are modeled to qualitatively discriminate the walnut shells in walnuts and whether mildew occurs using the SVM model. First, the K-S algorithm is used to divide 2445 sets of terahertz spectral data into a calibration set and prediction set, in which the number of spectra in the calibration set is 1834, and the number of spectra in the prediction set is 611. The four types of samples are numbered as “1”, “2”, “3”, and “4”. The grouped spectral data is then input into the SVM model, where the SVM type is c-svc, the penalty factor is c = 1, and the kernel function type is a linear (Liner) kernel function. Table 1 shows the classification results of the SVM discriminant model. The overall prediction number is 611, of which the number of correct predictions is 555, and the overall prediction accuracy reaches 90.83%. In a single category, the recognition accuracy of the model reaches 84.67%, 100%, 78.84%, and 100% for the walnut kernel, walnut shell, mildew samples, and reference group, respectively.

**Figure 5:**
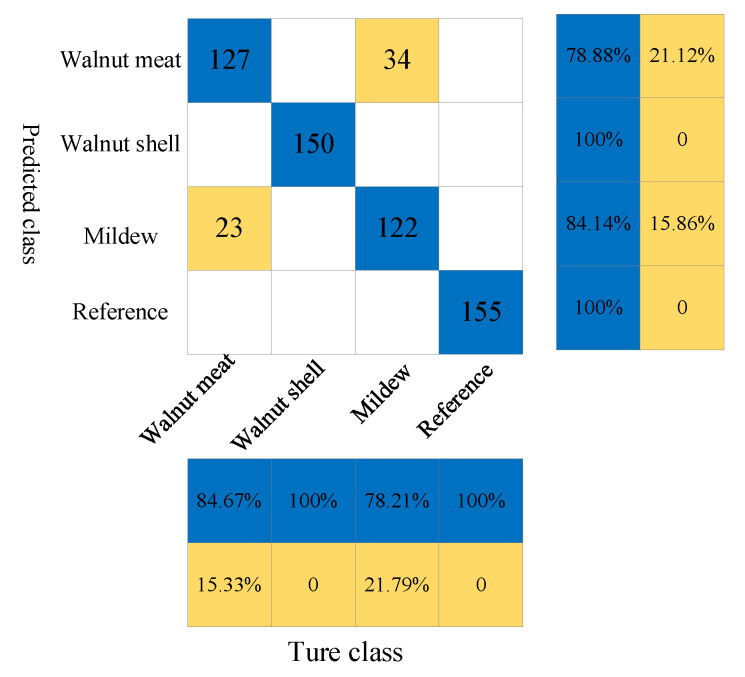
The confusion matrix of prediction results of walnut shell, walnut kernel, mildew sample and reference signal under SVM model.

In order to evaluate the prediction effect of the SVM model more intuitively, the predicted results of the prediction set are analyzed with a confusion matrix. As shown in Figure 5, there are 56 misjudgments among 611 samples, of which 23 walnut kernels are misjudged as mildew samples, and 34 mildew samples are misjudged as walnut kernels.

### 3.3. Establishment of Random Forest Qualitative Discriminant Model for Terahertz Spectra of Walnut Samples

**Figure 6:**
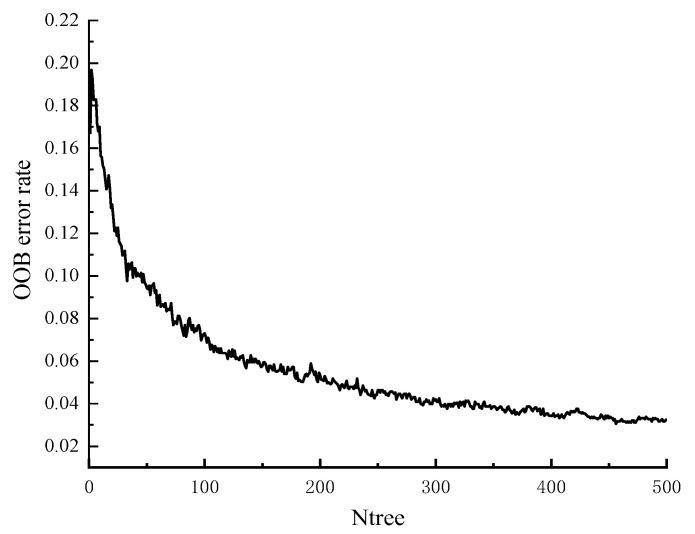
Relationship between OOB error rate and number of Ntree.

Similarly, the time–domain signals of 2250 THz bands in the full spectrum are modeled, and the random forest model is used to qualitatively discriminate the shells in the walnuts and the occurrence of mildew. First, the samples of the walnut kernel, walnut shell, mildew, and reference group are numbered as “1”, “2”, “3”, and “4”, respectively. Then, 2445 THz spectral data is randomly divided into a calibration set and prediction set according to 3:1 with a K-S algorithm. Among them, there are 1834 spectral data in the calibration set and 611 spectral data in the prediction set. Next, the data after grouping is input into the random forest model, and the relevant parameters are set, in which the number of decision trees (Ntree) is set to 500, and the number of point pre-selected variables (Mtry) is set to 47, followed by the construction of the random forest model. As shown in Figure 6, the out-of-bag (OOB) error rate is related to the number of decision trees. With the increase in the number of decision trees, the OOB error first decreases rapidly and then tends to be stable, and the minimum value of OOB error rate is 0.02187.

Table 2 shows the classification results of the random forest (RF) discriminant model. Compared with the SVM model, the prediction performance of the RF model has been further improved with 16 misjudgments in 611 prediction sets and an overall prediction accuracy of 97.38%. In the individual categories, the recognition accuracy of the model for the walnut kernel, walnut shell, mildew samples, and reference group is 92.67%, 100%, 96.79%, and 100%, respectively.

**Figure 7:**
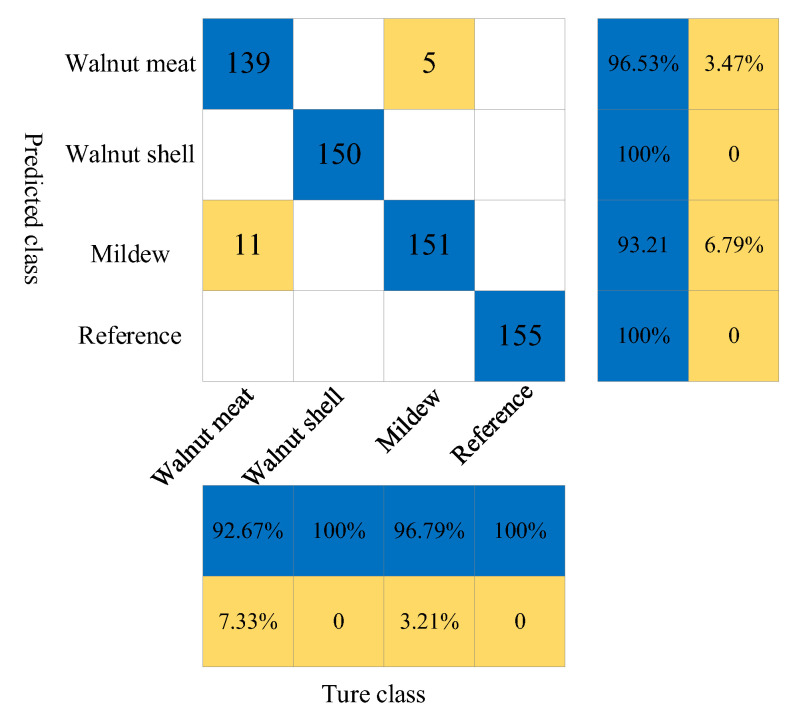
The confusion matrix of prediction results of walnut shell, walnut kernel, mildew sample and reference signal under RF model.

It can be seen from the confusion matrix in Figure 7 that there are 16 misjudgments in 611 samples, of which 11 walnut kernels are misjudged as mildew samples and 5 mildew samples are misjudged as walnut kernels, with a significant reduction in the number of misjudgments compared with the SVM model.

### 3.4. Establishment of the KNN Qualitative Discriminant Model for Terahertz Spectra of the Walnut Samples

The KNN model is used to model the time–domain signals of 2250 THz bands in the full spectrum to qualitatively discriminate the shells in the walnuts and the occurrence of mildew. First, the samples of the walnut kernel, walnut shell, mildew, and reference group are numbered as “1”, “2”, “3”, and “4”, respectively. Then, 2445 terahertz spectral data is randomly divided into a calibration set and prediction set in the ratio of 3:1 using the K-S algorithm, among which 1834 spectral data is in the calibration set and 611 spectral data is in the prediction set. Next, the data after grouping is input into the KNN model, and the nearest neighbor k is searched in the training data using a given distance metric. By comparing the four nearest neighbor numbers k, k = 1, 3, 5, 7, 9, 11, 13, 15, 17, 19, and 21, it is concluded that the KNN modeling effect is the best when k = 1, and the prediction accuracy reaches 97.87%.

Table 3 shows the classification results of the KNN discriminant model. It is found that the prediction accuracy of the KNN model is more accurate than that of the SVM and RF models. The prediction accuracy of both the walnut shell and reference group reaches 100%, and there are 9 and 4 misjudgments in the walnut kernel and mildew samples, respectively. In general, 598 samples of 611 prediction samples are correctly predicted, and the overall prediction accuracy reaches 97.87%, which is slightly higher than that of the RF model, and the prediction effect is the best in the three models.

**Figure 8:**
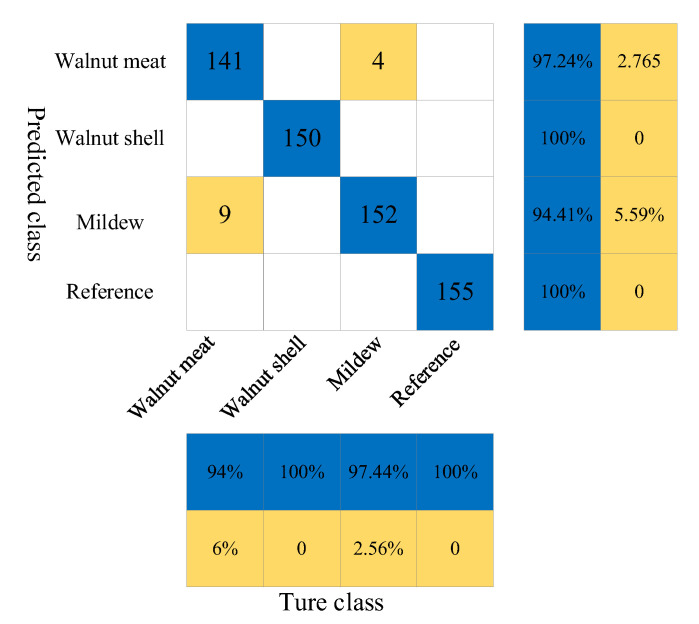
The confusion matrix of prediction results of walnut shell, walnut kernel, mildew sample and reference signal under KNN model.

As shown in the confusion matrix in Figure 8, there were 13 misjudgments in 611 samples, of which 9 walnut kernel data were misjudged as mildew samples and 4 mildew samples were misjudged as walnut kernel data. Through the analysis of the three confusion matrices, it is found that the misjudgment numbers are all from between the walnut kernel and mildew samples. This may be because the sample is not completely mildewed, or the spectrum of the walnut kernel is extracted when extracting the mildew region of interest.

### 3.5. Visual Expression of the Fullness of the Walnut Samples

#### 3.5.1. Fullness Detection of Terahertz Transmission Image

**Figure 9:**
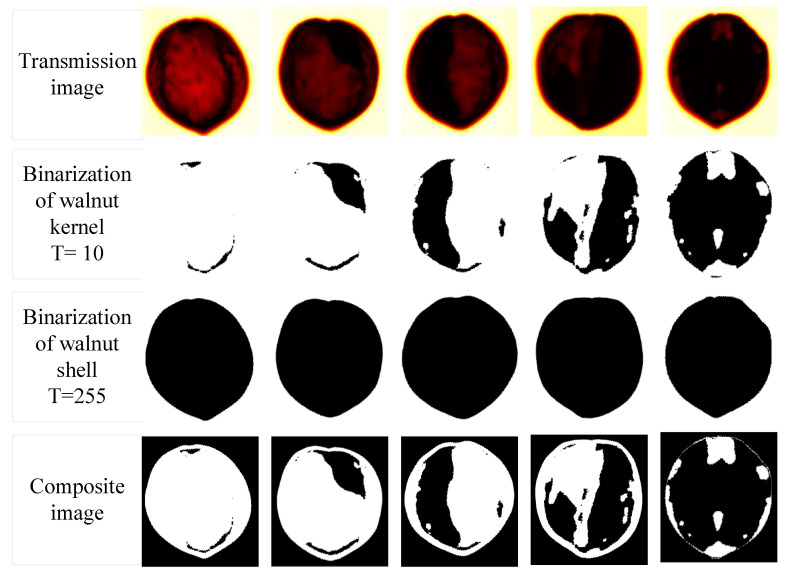
Terahertz transmission image processing flow of five types of samples with different plumpness.

Terahertz transmission imaging is performed on five kinds of kernel samples with different sizes in Figure 1, and the visualization expression of the walnut sample fullness is studied with the image processing method. Figure 9 is the THz transmission image processing flow of the walnut samples.

The first row in Figure 9 is the image of five types of samples under terahertz transmission imaging, the red area is the imaging area of the walnuts, and the black part of it is the kernel part. It can be seen from the image that the black area gradually increases from left to right, and it can be seen that the contour of the black area is similar to the shape of the walnut kernel in the original image, which is consistent with the placement rule, indicating that the fullness of walnuts can be characterized by the proportion of the black area in the red area in the image. The second row is the image after binarization of the kernels, and this step is realized using Matlab software with a set threshold of 10. It can be seen that the image after binarization processing becomes simpler and the contour of the target is better accentuated.

At this time, although the size of the kernel can be determined with manual observation, it cannot accurately determine the proportion of the walnut kernel in the walnut. The third row is the binarization of the shell, the threshold is set to 255, and the black area is the area of shell. The fourth row is the composite image of the shell and kernel. Through the composite image, the ratio of the kernel in the shell can be intuitively seen. The ratio of the pixels in the second and third row is the ratio of the kernel in the shell, which can be used to characterize the fullness of the walnut. Table 4 shows the walnut kernel fullness calculated with transmission images, which are 2.301%, 14.739%, 31.458%, 52.122%, and 83.762%, respectively.

#### 3.5.2. Fullness of Physical Images

**Figure 10:**
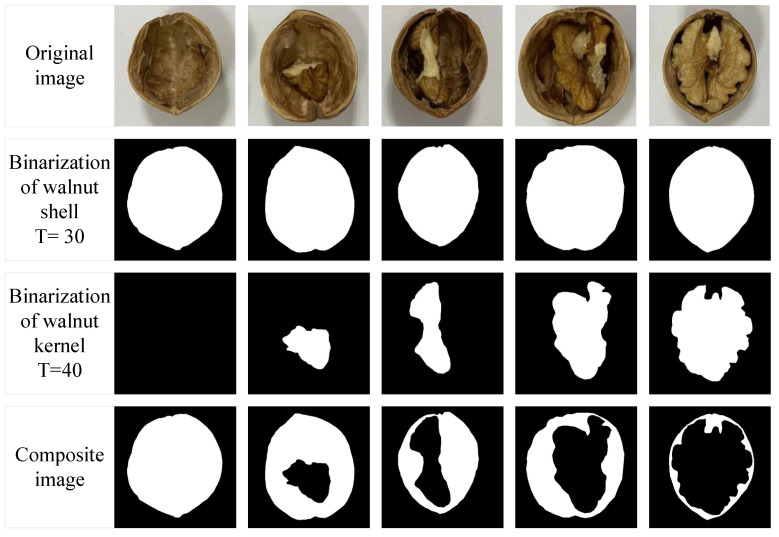
Original image processing flow of five types of samples with different plumpness.

Similarly, five walnut samples are arranged from small to large according to different kernels, and the fullness analysis is performed on the physical samples to facilitate the comparison with terahertz transmission imaging. Figure 10 shows the flow chart of the original image processing. The first row of Figure 10 shows the processed five types of walnut samples with gradually increasing kernels from left to right, and these five original images are processed according to the same processing method. The second and third rows in Figure 10 are the images after binarization processing of the walnut shell and walnut kernel, in which the threshold value set for walnut shell processing is 30 while the threshold value set for walnut kernel processing is 40.

Through binarization, it can be seen that the edge curves of the processed images are smooth, and the shapes are basically the same as that of the original images, which can well characterize the contours of the walnut shells and walnut kernels. The fourth row is the combined images of the walnut shell and walnut kernel after processing; the white area is the area of the walnut shell, and the black area inside the white area is the area of the walnut kernel. The combined image reflects the fullness of the walnut kernel. Similarly, the proportion of walnut kernels in the walnut shell of the original image is calculated, and the calculation results are shown in Table 5, which are 0%, 18.949%, 34.496%, 51.483%, and 80.227%.

#### 3.5.3. Fullness Comparison between Physical Image and Transmission Image

Table 6 shows the walnut kernel fullness of the transmission images and the physical images. The fullness information of the transmission image in Table 4 and the physical image in Table 5 are summarized in the same table for convenient comparison. It can be seen from Table 6 that the maximum difference between the fullness of five walnuts with different kernel sizes is no more than 5% in their transmission images and physical images. Considering the complexity of the walnut samples, the error is within the allowable range and does not affect the evaluation of the walnut quality.

## 4. Conclusions

In this paper, terahertz transmission imaging technology is used to identify and analyze normal walnuts and mildewed walnuts while image processing is performed on physical samples with different kernel sizes to calculate the fullness of the walnuts. First, the image information of the walnuts is collected, and the terahertz spectral information for different regions of interest is extracted. Three qualitative discrimination models of SVM, RF, and KNN are established, and the overall identification accuracy of the three models reaches 90.83%, 97.38%, and 97.87%, respectively. The KNN has the optimal qualitative discrimination effect. Among them, in a single category, the recognition accuracy of the model for the walnut kernel, walnut shell, mildewed samples, and reference group reaches 94%, 100%, 97.43%, and 100%, respectively. The terahertz transmission images of five kinds of physical samples with different kernel sizes are processed, and the fullness of the walnuts is characterized by calculating the ratio of the number of pixels of the walnut kernels to walnut shells in the binary images. Meanwhile, the original images of the samples are also processed, and their fullness is calculated. It is found that both errors are less than 5%, indicating that terahertz imaging technology combined with image processing can effectively evaluate the fullness of the walnut. In this case, this study provides a non-destructive method for walnut mildew detection, and it also provides a new method to analyze the fullness of walnut kernels, which has important value in practical application. Terahertz spectroscopy and imaging provides a non-destructive detection method for walnut quality, which can provide a reference for the quality detection of other dried nuts with shells, thus having significant practical value.

## Figures and Tables

**Table 1:** Classification results of SVM discriminant model.

Samples	Cal	Val	Category	Prediction	Correct	Accuracy
2445	1834	611	Walnut Kernel	150	127	84.67%
			Walnut Shell	150	150	100%
			Mildew Sample	156	123	78.84%
			Reference Group	155	155	100%
2445	1834	611	Total	611	555	90.83%

**Table 2:** Classification results of RF discriminant model.

Samples	Cal	Val	Category	Prediction	Correct	Accuracy
2445	1834	611	Walnut Kernel	150	139	92.67%
			Walnut Shell	150	150	100%
			Mildew Sample	156	151	96.79%
			Reference Group	155	155	100%
2445	1834	611	Total	611	595	97.38%

**Table 3:** Classification results of KNN discriminant model.

Samples	Cal	Val	Category	Prediction	Correct	Accuracy
2445	1834	611	Walnut Kernel	150	141	94%
			Walnut Shell	150	150	100%
			Mildew Samples	156	152	97.44%
			Reference Group	155	155	100%
2445	1834	611	Total	611	598	97.87%

**Table 4:** Calculation results of walnut kernel fullness by walnut transmission images.

Category	Walnuts (a)	Walnuts (b)	Walnuts (c)	Walnuts (d)	Walnuts (e)
Kernel Total Pixels	191,229	197,756	214,005	206,360	210,020
Shell Total Pixels	4401	29,147	67,321	107,558	171,068
Fullness	2.301%	14.739%	31.458%	52.122%	81.453%

**Table 5:** Fullness of walnut kernel in physical image.

Category	Walnuts (a)	Walnuts (b)	Walnuts (c)	Walnuts (d)	Walnuts (e)
Kernel Total Pixels	125,054	126,708	103,295	130,339	113,698
Shell Total Pixels	0	24,010	35,633	67,103	91,216
Fullness	0%	18.949%	34.496%	51.483%	80.227%

**Table 6:** Fullness of walnut kernels in transmission and physical images.

Category	Walnuts (a)	Walnuts (b)	Walnuts (c)	Walnuts (d)	Walnuts (e)
THz Image Fullness	2.301%	14.739%	31.458%	52.122%	81.453%
Kernel Fullness	0%	18.949%	34.496%	51.483%	80.227%
Error	2.301%	4.210%	3.038%	0.639%	1.226%

## Data Availability

The data presented in this study are available on request from the corresponding author. The data are not publicly available due to privacy.
